# The Vienna Statement; an Update on the Surgical Treatment of Sportsman's Groin in 2017

**DOI:** 10.3389/fsurg.2018.00045

**Published:** 2018-07-04

**Authors:** Aali J. Sheen, J. James Pilkington, Moshe Dudai, Joachim K. Conze

**Affiliations:** ^1^Manchester University Foundation Trust, Manchester, United Kingdom; ^2^Centre for Biomedicine, Manchester Metropolitan University, Manchester, United Kingdom; ^3^Hernia Excellence, Ramat Avic Medical Center, Tel Aviv, Israel; ^4^Hernienzentrum, Munich, Germany

**Keywords:** sportsman's hernia, inguinal disruption (ID), inguinal hernia (IH), pubic inguinal pain syndrome (PIPS), athletic pubalgia, inguinal ligament release

## Introduction

The definition of the popularly termed “Sportsman's Groin” (SG) was recently addressed in two consensus meetings (1, 2). These peer-reviewed agreements decided upon the terms “Inguinal Disruption” (ID) and “Inguinal related pain” as terminologies to describe groin pain in an athlete where no true hernia exists (1, 2). Other terms used include; “Athletic Pubalgia,” “Incipient Hernia,” “Gilmore's Groin,” and “Pubic Inguinal Pain Syndrome” (PIPS) (3).

Controversy still remains as to the exact origin of the pain experienced including the pathology; groin related, adductor related, inguinal related, hip related or most likely a combination of multiple pathologies (4, 5)? Management strategies include the use of physiotherapy to improve core stability, exercise to facilitate adductor strengthening and other non-operative measures such as plasma enriched protein (PRP) and steroid injections (6, 7).

Following clinical assessment and exclusion of other pathology with the use of magnetic resonance imaging (MRI) (8), surgery to the inguinal canal has been recommended (9). Both open and laparoscopic [laparoscopic trans-abdominal pre-peritoneal (Lap TAPP) and laparoscopic totally extra-pre-peritoneal (Lap TEP)] repairs techniques have been utilized (10, 11) with no significant differences being reported to date (12). The inguinal ligament release procedure (13) and the open minimal repair (OMR) (11) were selected as the operation of choice.

To the author's knowledge, this is the first time that a number of specialized surgical techniques have been explored in an open forum.

This paper summarizes the opinions obtained through a session held at the 39th Annual Congress of the European Hernia Society (EHS) in Vienna designed to explore opinion and current practice for surgical management of “Sportsman's Groin” and “Inguinal Disruption” within the surgical community.

## Materials and methods

A session was organized at the 39th International Congress of the EHS meeting to question and inform delegates on the contemporaneous surgical treatment of ID (1, 2). Chairs and speakers were selected for their proven record of accomplishment and experience in this field. Speakers were given a structure to follow in their presentation (see Appendix 1).

Participants were asked to complete a series of background questions (see Appendix 2).

Prior to the presentations, the delegates were asked four main debate questions (see Appendix 3).

Once responses had been collected, the following presentations were given:

Prof. David Lloyd-TAPP Release
  A novel treatment designed to relieve the tension in this area by releasing the inguinal ligament, conjoint tendon and lateral rectus sheath and reinforcing the whole area with a non-reactive mesh.

Dr. Ulrike Muschawek–Open Minimal Repair (OMR)
  A repair involving division and subsequent reconstruction of the transversalis fascia under local anesthesia using a simple non-absorbable continuous suture from the pubic tubercle to the deep inguinal ring, with or without division of the genital branch of the genitofemoral nerve. No mesh is used.

Prof. Moshe Dudai-TEP release and Reinforce
  Surgery involves the release of pathological adhesions within the inguinal canal and the reduction of a herniated lipoma. A mesh prosthesis is used.

Dr. Andreas Koch-Open Maximal Repair (Meyer's mod.)
  A variation on the minimal repair with deeper placement of a reinforcing suture that includes the lacunar ligament. Described as a “Meyer's modification.” Only 13% of patients seen proceed to surgery.

Prof. Hannu Paajanen-TEP Reinforce
  Lap TEP approach to site a lightweight 15 by 12 cm mesh without the use of mesh-fixation methods.

Commonality in the presentations were achieved in the following areas

1) Clinical examination is a dominant factor in the diagnosis
2) There is pathology in the posterior wall
3) There is pathology in the Inguinal Ligament
4) There is a need for reinforcing the posterior wall
5) A post-operative “Adapted Athletic Rehabilitation Program” is recommended

Delegates were asked to answer the four main debate questions again.

Data collected throughout the session was collated and analyzed.

### Statistics

The individual responses for background information were explored with descriptive statistics (frequencies, percentages) and cross-tabulations. To compare the type of surgery used for inguinal hernia and inguinal disruption the proportion of agreement was calculated with 95% binomial confidence intervals and Cohen's Kappa was obtained, comparing agreement between the two conditions.

For the debate questions, which were only available summarized by question, the proportions giving each response with 95% binomial confidence intervals were calculated.

All analyses were undertaken in R version 3.0.2 (14).

## Results

Sixety-Seven delegates (approx. 45%) responded. The audience demographics: General surgeons (58%) and Specialist Hernia surgeons (13%). Forty-Six percent of the surgeons were 45–60 years age range. There was a high level of experience with a considerable number having practiced for over 15 years (40%).

The commonest surgery undertaken for IH was Lap TAPP (46%) followed by Lap TEP (37%). Forty-Five percent reported the use of minimal access techniques for the majority of their practice. For IH 1.5% respondents use the OMR. For ID; Lap TAPP (43%), Lap TEP (37%), and OMR (9%) were used. This indicates an increase in the number of respondents choosing OMR for ID compared with IH.

A minority of respondents (7%) reported that they saw over fifty cases of SG per year with 67% seeing less than 10 cases per year.

A large majority of the respondents (83%) reported to undertaking some form of preoperative investigation in the management of ID. When confident in the diagnosis; some (13%) proceed straight to surgery, and most (85%) recommend another treatment modality first; physiotherapy (47%), rest and analgesia (38%), a trial of steroid injections (2%).

Some surgeons will undertake bilateral ID repair for a patient presenting with unilateral signs (15%).

Most surgeons would not be willing to undertake an adductor tendon release at the same time as an ID repair (85%).

Return to sporting activity ranged across the room; 2 weeks (18%), 2–4 weeks (34%), 4–6 weeks (42%).

Forty of the Sixety-Seven respondents (60%) had complete individual level raw data available. Data from 27 delegates that used the electronic submission was unfortunately not made available by the conference organizers. Of the 40 respondents, most performed Lap TEP or Lap TAPP for IH repair (92.5%) and ID repair (87.5%). For IH repair the remainder used an open mesh repair. For ID repair other methods included OMR (1), Open-Darn (2), Open Mesh (1), and Release (1).

Generally, the operation undertaken for an IH did not differ significantly from the surgery offered by each individual surgeon for ID. For 31 participants the type of surgery agrees for both IH and ID. This is equivalent to 77.5% (61.5–89.2). Using a Cohen's un-weighted Kappa test to examine for agreement between surgeries for the two types of hernia, accounting for chance, an estimate of 0.62 (95% CI 0.44–0.81) is given. This indicates a moderate to substantial agreement between the two groups of operations.

The numbers who responded before and after varied on and between each question (Table 1). All interpretation is at a basic level and percentages have wide confidence intervals.

**Table 1:** A summary of debate questions before and after presentations and debate.

**Question**	**Response**	**Before debate**		**After debate**	
		**N**	**Percentage (95% CI)**	**N**	**Percentage (95% CI)**
1	Total	41		21	
	SG	26	63.4(46.94−77.88)	9	42.86(21.82−65.98)
	ID	3	7.3(1.54−19.92)	1	4.8(0.12−23.82)
	IRP	5	12.2(4.08−26.20)	3	14.3(3.05−36.3)
	AP	0	0.0(0.00−8.60)	2	9.5(1.17−30.38)
	PIPS	7	17.1(7.15−32.06)	6	28.6(11.28−52.18)
2	Total	46		37	
	Surgery	6	13.0(4.94−26.26)	3	8.1(1.70−21.91)
	Physio	17	37.0(23.21−52.45)	15	40.5(24.75−57.90)
	Rest	21	45.7(30.90−60.99)	18	48.6(31.92−65.60)
	PRP/other	2	4.3(0.53−14.84)	1	2.7(0.07−14.16)
3	Total	49		41	
	TEP	10	20.4(10.24−34.34)	11	26.8(14.22−42.94)
	TAPP	17	34.7(21.67−49.64)	5	12.2(4.08−26.20)
	Open mesh	5	10.2(3.40−22.23)	0	0.0(0.00−8.60)
	OMR	10	20.4(10.24−34.30)	10	24.4(12.36−40.30)
	TAPP-Rel	4	8.2(2.27−19.60)	10	24.4(12.36−40.30)
	TEP-Rel	3	6.1(1.28−16.87)	5	12.2(4.08−26.20)
4	Total	54		43
	Yes	26	48.1(34.34−62.16)	17	39.5(24.98−55.59)
	No	20	37.0(24.29−51.26)	21	48.8(33.31−64.54)
	DK	8	14.8(6.62−27.12)	5	11.6(3.89−25.08)

For question 1 “Definition” there is some suggestion that the proportions do change after the debate with fewer appearing to select “Sportsman's Groin” (63–43%) and more selecting “Pubic Inguinal Pain Syndrome” (17–29%).

For question 2 “Main treatment” there is a greater level of response but overall we see little change in answers after the debate.

Question 3 “Surgical Choice of repair” demonstrated a change in approach with increased response for TEP (20–27%), TAPP–Release (8–24%), and TEP–Release (6–12%).

Question 4 “Is mesh essential” shows a decrease in the numbers of “don't know” (15–5%) and “yes” (48–40%). This might suggest that more respondents were convinced by the notion of a mesh-less or suture only repair after the debate.

When comparing pre-debate answers with post-debate answers we see a leaning toward PIPS as the definition and that delegates were influenced against the notion that placement of mesh is an essential component of the surgery for “Sportsman's Groin.”

## Discussion

Surgeons that see such patients with a diagnosis of “Sportsman's Groin” mainly have a specialized interest in this field with various surgical intervention described (12) and anecdotally there is some evidence that patients are provided with good results. Consensus statements have established some guidance for the investigation and management of Sportsman's groin (1) as well as the possible contributory pathology (2). There is still great heterogeneity in academic research and clinical practice and this opinion paper set out to address key issues specific to the surgical management of “Sportsman's Groin.”

Pain in the groin can be thought of as a repetitive strain injury (15). The pain experienced can be multifactorial (44%) and the majority will have some pain related to the adductor muscles (61%) (5). The Doha statement used the Delphi methodology and identified three main causes of groin pain; (1) Groin related (adductor-related, inguinal-related, pubic related or ilio-psoas related); (2) Hip-related, or (3) other causes (2). Following both the Manchester and Doha statements (1, 2) the definition of “Inguinal disruption” (ID) or “Inguinal related groin pain” were respectively decided upon for a “Sportsman's hernia.”

The results from the audience showed a leaning toward “Pubic inguinal pain syndrome” (PIPS), which although not a significant trend, potentially rejects the Manchester and Doha definitions.

A previous survey of European surgeons has drawn no conclusions (16). Laparoscopic repair has been shown in a randomized trial to provide significant benefit in comparison to a physiotherapy program (17, 18). It is important to highlight that until now no randomized trial has been undertaken to compare a sutured repair with a laparoscopic technique for the treatment of “Sportsman's groin” (NCT01876342) (19).

No consensus report has discussed why there is a disparity in the chosen repair technique.

Respondents did not appear willing to perform adductor release procedures although low-level evidence has shown its safety and efficacy in the treatment of adductor enthesopathy in athletes (20).

Speakers presented their personal understanding of the pathology involved with “posterior wall weakness”; a common finding on ultrasonography that has also been found in asymptomatic individuals (1). It was agreed that the concept of “posterior wall weakness” as a contributing factor to the pain does need further definition and exploration.

All described the concept of an early return to sporting activity. One speaker stating that 2 weeks is sufficient post-operative recuperation in the case of an elite footballer (soccer player). A claim that goes unsubstantiated with any report or data.

The debate was structured around “a player” with isolated groin pain. Each surgeon was allowed to present their respective case. In the context of “a player,” we have summarized some of the findings from this meeting:

What is the most popular choice of repair and does any particular injury change the treatment plan for the patient?
  Laparoscopic repair, whether TEP or TAPP, was overwhelmingly the most common repair for both ID (80%) and IH repair (83%).
  Do most surgeons undertake the same repair as for inguinal hernias and sports hernias or are there any differences noted.
  Before the debate, surgeons generally reported to undertaking the same repair for both IH and ID (Kappa un-weighted coefficient 0.62).
  Based on the talks did delegates change their opinion on the type of repair they would perform?
  There appeared to be a move toward the inguinal release procedures and OMR; a view becoming popularized in the literature (11, 13).
  Is mesh essential?
  There appeared to be a move away from the mesh as an essential component of this repair.

## Limitations

It is accepted that the concept of this manuscript relies on subjective opinion of leading experts which opens it to a high levels of bias and scrutiny. In addition, the responses are limited to the individuals that were able to attend and they were not multidisciplinary.

Further caution must be undertaken in interpretation of the data as it was difficult to determine the exact answers pre and post-debate for all the questions and like all question and answer based surveys, response was less than ideal.

## Conclusion

This debate shows and confirms that surgeons are likely to undertake the same operation to repair ID as they would IH.

## Author contributions

AS: design of meeting, questions, analyses of data, and writing of manuscript; JP: editing of manuscript and data analyses; MD and JC: design of meeting and questions as well as manuscript.

### Conflict of interest statement

The authors declare that the research was conducted in the absence of any commercial or financial relationships that could be construed as a potential conflict of interest.
