# Supplementary material for: The Vienna Statement; an Update on the Surgical Treatment of Sportsman's Groin in 2017
Source: Front Surg. 2018 Jul 4;5:45. doi: 10.3389/fsurg.2018.00045 (PMC6043792; doi:10.3389/fsurg.2018.00045)
Supplement: Supplementary file 1 [file Data_Sheet_1.pdf]

Appendix

Appendix 1 – Key points for discussion that were given to the five key speakers prior to the meeting.

- Describe the specific pathology that you are treating with an intended surgical repair
- Provide the details of the procedure: surgical steps with video clips or pictures
- What is your operation designed to achieve, to deal with or resolve the specific pathology?
- What are the advantages of your approach? Why did you select it?
- What are the disadvantages of your approach?
- What management do you prescribe for patients with a combined pathology of a 'Sportsman Hernia' and 'Athletic Pubalgia' or do you treat these entities in the same manner?
- Describe your post-operative Sport Muscles Rehabilitation Program? Do you adapt it to the severity of the injury?
- When do you expect your patients to start rehabilitation after their surgery?
- What is the average time your patients take to return to their chosen sport after surgery and rehabilitation?

Appendix 2 – Audience pre-session questionnaire

Demographics

Country or origin ……………………..

a) EU
b) Outside EU or Africa
c) Asia

Type of surgeon

a) General Surgeon
b) Specialist hernia surgeon
c) Cancer surgeon with hernia interest
d) Non cancer surgeon with hernia interest
e) General surgeon with no hernia specialist interest

Your Age

a) 25-35
b) 35-45
c) 45-60
d) 60+

Years of experience as an independent practitioner

a) 0-5 years
b) 5-10 years
c) 10-15 years
d) 15+ years

What percentage of your regular daily surgical procedures do you undertake Laparoscopically

a) <10%
b) 10-30%
c) 30-50%
d) >50%

In your routine practice for a Standard inguinal hernia, by what method do you undertake the repair?

a) Lap TEP
b) Lap TAPP
c) Open Mesh
d) Open Tissue Repair
e) Open darn
g) Other - please specify

1) How many sports hernias do you see per year

a) <10
b) 10-25
c) 25-50
d) >50

2) What imaging modalities do you use in your assessment of SH patients (tick all that apply)

a) None clinical examination only
b) Dynamic ultrasound
c) MRI pelvis
d) Dexa Scan

3) Once a diagnosis of a sports hernia has been made what is your preferred initial treatment

a) Straight to surgery
b) Surgery after one conservative trail
b) Physiotherapy
c) Analgesia and rest
d) other - please specify

4) If you feel that surgery is warranted in your opinion which operation are you most likely to perform

a) Lap TEP
b) Lap TAPP
c) Open Mesh
d) OMR
e) TAPP or TEP Release
f) open darn
g) Other - please specify

5) Do you routinely undertake bilateral repair even for unilateral signs?

a) Yes

b) No

6) If required would you also undertake an adductor tenotomy?
a) Yes
b) No

7) After surgery what period of time do you expect on average your patient to return to normal or sporting activities?
a) 2 weeks
b) 2-4 weeks
c) 4-6 weeks
d) >6 weeks

Appendix 3 – Debate questions used for the session

1) What is your preferred definition of a painful groin in an athlete
    a. Sportsman's groin
    b. Inguinal Disruption
    c. Inguinal related groin pain
    d. Athletic pubalgia
    e. Pubic inguinal pain syndrome
2) Once a diagnosis of inguinal disruption is made what is your mainstay of treatment
    a. Straight to surgery
    b. Physiotherapy
    c. Analgesia and rest
    d. PRP or other treatment e.g. massage
3) If you consider surgery what operation will you perform
    a. TEP
    b. TAPP
    c. Open Mesh
    d. OMR
    e. Open Release
    f. Lap Release
4) Do you consider the use of mesh essential in any repair
    a. Yes
    b. No
    c. Don't know
